# The Nature of Malignant Testicular Tumours

**Published:** 1909-02-13

**Authors:** 


### THE NATURE OF MALIGNANT TESTICULAR TUMOURS.

Until quite recently the generally accepted opinion upon malignant tumours of the testicle has been that they are either primary sarcomata or primary carcinomata; and most observers have held that the sarcomata are the more numerous. This view needs considerable modification, as the interesting work carried out by Dr. G. W. Nicholson in the Pathological Museum at Guy's Hospital shows. The full details of this work are to be found in a paper in the "Guy's Hospital Reports" for 1907. Perhaps the most interesting of all the points he brings out, is the fact that a large number of testicular tumours are directly comparable to certain ovarian neoplasms, in that they contain derivatives not of epiblast only, nor of hypoblast only, nor of mesoblast only, but of all these three primary embryonic layers. The tumour is, in a word, a particular variety—an Embryoma.

Dr. Nicholson's general conclusions, based upon 65 specimens, are as follows:—

1. Spheroidal-celled carcinoma of the testicle is in nearly every case an encephaloid cancer. It may be alveolar or non-alveolar. It is often mistaken for sarcoma. It may occur at an early age, and runs a clinically malignant course. Next to embryoma, it is the commonest form.

2. Sarcoma is usually, if not always, composed of round cells. It occurs at a somewhat earlier age than carcinoma, and is even more malignant. It is far less common than is generally supposed. In the young it may be bilateral.

3. There is evidence to show that carcinoma disseminates mainly by the lymphatics, and sarcoma by the blood-vessels. It was formerly taught that testicular sarcoma, unlike sarcoma in general, was particularly prone to infect lymphatic glands. This view was doubtless due to misinterpretations of the histological characters of the tumours; for many which were really carcinomata, or embryomata, were miscalled sarcomata.

4. Endothelioma may arise in the endothelium of the blood-vessels or lymphatics. It occurs usually in young adults, but may do so at any age. Although it generally runs a relatively benign course, it may be very malignant and produce secondary deposits in the lymphatic glands.

5. Embryoma is the commonest new growth of the testicle, but it is often overlooked. Although not necessarily malignant, it may produce metastases composed of all the tissues of the primary growth; or one tissue only may become malignant, in which case the deposits consist of that tissue only, and therefore simulate carcinoma, or sarcoma.

6. Some embryomata contain trophoblast, which can be shown to have originated in the epiblast of the primary tumour. In this case the trophoblast assumes malignant characters, the metastases being composed of the cells of this tissue alone. These tumours are very malignant, and usually occur in young adult life.

The essential feature of an embryoma is that it exhibits derivatives of more than one of the primary embryonic layers, and in a typical case exhibits derivatives of all three—epiblast, mesoblast, and hypoblast. The following is an instance in point:—

A youth of nineteen died within a few months of the removal of a testicular tumour. At the post-mortem examination the liver was found to contain a single deposit of new growth, and the lumbar glands were infiltrated. Microscopically the growth showed a stroma represented by thin trabeculæ of connective tissue which in places became extremely cellular and resembled embryonic connective tissue. This stroma was clearly mesoblastic. The epiblast was represented by islands and strands of stratified epithelium, whilst the main mass of the growth was composed of tissue resembling columnar-celled carcinoma, clearly hypoblastic in origin.

Although in such a tumour derivatives of all three embryonic layers are to be found, it is noteworthy that when malignant characters arise in it, it by no means follows that all the elements of an original embryoma will take on equally malignant characters. An embryoma is by no means essentially malignant; when it becomes so, the epithelial elements may preponderate so that carcinoma is simulated unless several sections are examined; or else the connective tissue elements may preponderate and sarcoma may be diagnosed incorrectly. Again, the epithelial and the connective tissue elements may be proliferated about equally. Thus the microscopic characters of embryomata vary widely, both in different cases and in different parts of the same specimen.

That an embryoma is not essentially malignant is exemplified by the case of a man of 47 who had had an enlarging testicle for a month and then had it removed. The mass was some four times the natural size of a testis and contained numerous cysts. Histologically representatives of all three embryonic layers were found. The epithelial cells looked natural, however. That this embryoma was relatively benign was further indicated by the fact that the man survived his operation for ten years, and then died not of recurrence, but of a railway accident.

Dr. Nicholson was able to demonstrate in different testicular embryomata the following derivatives of the various embryonic layers:—

1. Hypoblastic: Columnar epithelial columns or cysts, some with goblet cells like those of the normal bowel, others with ciliated margins like those of the respiratory tract.

2. Mesoblastic: Cellular connective tissue like that of an embryo; myxomatous tissue; bone; lymphatic tissue; striped muscle fibre; hyaline cartilage; smooth muscle fibre.

3. Epiblastic: Stratified epithelium either as solid plugs, or as the lining of larger or smaller cysts; nerve matter and ganglion cells; teeth; and a rudimentary retina.

An embryoma is not necessarily present at birth. At least, the clinical signs of it are not to be detected at that time; the embryoma shows itself as a swelling of a testicle that has previously seemed quite natural. Of 16 cases, the oldest was 58, the youngest a year and ten months, the average age at the time of operation being very nearly 30.

